# The Blondex Palatal TAD-Supported Distalizer: A Technical Report of Three Independent Clinical Cases Using Analog, Mixed, and Fully Digital Workflows

**DOI:** 10.3390/healthcare14193184

**Published:** 2026-09-25

**Authors:** Matteo Saccucci, Guglielmo Biondi, Alex Bruno, Aurora Pasqualini, Gabriele Di Carlo

**Affiliations:** 1 Department of Oral and Maxillo-Facial Sciences, Sapienza University of Rome, Viale Regina Elena 287a, 00161 Rome, Italy; aurora.pasqualini@uniroma1.it; 2 Private Practice, 36100 Vicenza, Italy; drguglielmobiondi@gmail.com; 3 Nexxta-Ortotec, 33100 Udine, Italy; bruno.alex.priv@gmail.com; 4 BeSSA Department, Wellbeing, Health and Environmental Sustainability, Sapienza University of Rome, Via della Fontanelle, 02100 Rieti, Italy; gabriele.dicarlo@uniroma1.it

**Keywords:** TADS, molar distalization, digital planning in orthodontics

## Abstract

**Background:** Maxillary molar distalization is frequently used for Class II correction, but conventional appliances may be associated with compliance requirements and unwanted dental side effects. Skeletal anchorage can improve anchorage control and facilitate more targeted posterior tooth movement. **Aim:** To describe the design, biomechanical rationale, and clinical application of the Blondex palatal TAD-supported distalizer through three illustrative clinical cases managed via different clinical and laboratory workflows. Detailed case description: Three patients requiring maxillary molar distalization were treated with the Blondex appliance supported by two palatal miniscrews. The cases illustrate an analog workflow based on conventional impressions and freehand miniscrew placement, a mixed workflow using digital diagnostic planning with hybrid laboratory procedures, and a fully digital “one-visit” workflow integrating CBCT, intraoral scans, CAD/CAM surgical guidance, miniscrew placement, and appliance delivery. In all three cases, the appliance was used to establish Class I molar and canine relationships while incorporating control over molar derotation, distalization, and, where indicated, vertical movement. Because of the descriptive design and the heterogeneity of the cases, no pooled estimate of distalization rate or treatment effect was calculated. **Conclusions:** This three-case series illustrates the clinical versatility of the Blondex appliance and its compatibility with analog, mixed, and fully digital workflows. The appliance may be considered in selected patients requiring TAD-supported maxillary molar distalization, but its comparative effectiveness, skeletal and dental side effects, and long-term stability require evaluation in larger controlled studies.

## 1. Introduction

### Literature Analysis and State of the Art

Correction of Class II malocclusions aimed at gaining arch space or improving the molar relationship often requires distal movement of the maxillary molars when a non-extraction treatment approach is selected. Traditionally, this objective has been achieved by using extraoral appliances, such as headgear, or intraoral distalization devices, including the Pendulum and Distal Jet appliances. However, these treatment modalities present important limitations: their effectiveness is frequently dependent on patient compliance, particularly in the case of extraoral traction [1,2,3,4,5,6], and they are often associated with anchorage loss, resulting in undesirable side effects such as mesial movement of the anchorage unit and proclination of the maxillary incisors [7]. The introduction of temporary anchorage devices (TADs) has significantly changed the biomechanics of maxillary molar distalization. By providing skeletal anchorage that is largely independent of the dentition, TADs minimize anchorage loss and allow more efficient distal movement of the posterior segments while maintaining greater control over the anterior teeth. As a result, unwanted side effects, such as maxillary incisor proclination and mesial migration of the anchorage unit, can be substantially reduced [8,9]. Conversely, miniscrew-supported appliances may offer limited versatility when used in complex clinical scenarios. The anterior palate has emerged as the preferred site for orthodontic miniscrew insertion because of its favorable anatomical characteristics. This region offers adequate bone volume and density, particularly in the median and paramedian areas, ensuring excellent primary stability [10,11]. Furthermore, the absence of adjacent dental roots minimizes the risk of root damage during insertion [12]. The overlying palatal mucosa is thin and highly keratinized, which facilitates oral hygiene maintenance and is associated with a lower prevalence of peri-implant inflammation compared with buccal insertion sites [13]. This article aims to illustrate the structural and functional characteristics of the Blondex orthodontic appliance and to describe the design, biomechanical rationale, and clinical application of the Blondex palatal TAD-supported distalizer through illustrative clinical cases managed via different clinical and laboratory workflows.

## 2. Detailed Case Description

### 2.1. Study Design and Case Selection

This manuscript reports a descriptive case series of three patients treated with the Blondex appliance for maxillary molar distalization. The cases were selected to illustrate three different methods of diagnosis, appliance fabrication, miniscrew positioning, and clinical delivery: an analog workflow, a mixed workflow, and a fully digital “one-visit” workflow. The cases were not intended to constitute a comparative clinical trial or to provide an estimate of the average treatment effect of the appliance.

All patients were treated in a private orthodontic practice in Vicenza, Italy. The clinical indications for distalization, the dentition stage, the sagittal and vertical relationships, and the treatment objectives were assessed individually. Written informed consent for treatment and publication of clinical records and images was obtained from all patients or their legal guardians.

### 2.2. Technical Features: The Blondex Appliance

The Blondex appliance was developed to apply distalization mechanics anchored to palatal miniscrews, overcoming the limitations of conventional devices that often induce mesial rotations and offer limited versatility. The Blondex is a distalizing device anchored to two or three palatal miniscrews and two bands on the upper first molars. It features a rail system along which activators slide, connected to helices or springs made of TMA or stainless steel. Activation is achieved through a latch mechanism that loosens the locking screw, allowing the activator to move. The distal displacement of the activator generates an elastic force that activates the spring, generally a round 0.032-inch TMA wire. Horizontally oriented TMA springs promote molar derotation and control tipping, while vertically oriented stainless steel springs produce distalization (Figure 1). The appliance allows both unilateral and bilateral distalization.

In its original design, the device featured two round sliding arms with soldered cannulas to control unwanted rotations (Figure 2). The introduction of a square wire eliminated these issues, enhancing movement stability and precision (Figure 3).

## 3. Clinical Assessment

The cases were documented using extraoral and intraoral photographs, panoramic radiographs, lateral cephalograms, and, where applicable, digital scans and CBCT-based planning. Treatment duration and the achievement of the intended occlusal objectives were recorded descriptively. No inferential statistical analysis was performed because of the small sample size, the heterogeneity of the cases, and the descriptive objective of the report. Post-treatment CBCT was not routinely obtained solely for research purposes, in accordance with the ALARA principle; therefore, three-dimensional assessment of root position, cortical bone contact, and root resorption was not possible.

Clinical evaluation of the Blondex appliance confirmed its technical feasibility and versatility across three distinct execution workflows (Table 1).

Analog workflow: Represents the conventional approach, relying on traditional physical impressions, master plaster models, and freehand TAD placement. This workflow validates the basic biomechanical setup of the Blondex appliance without requiring complex digital hardware.

Mixed workflow: Combines digital diagnostic planning (such as 2D digital cephalometric analysis or 2D surgical templates) with hybrid laboratory procedures. It offers an intermediate solution that streamlines laboratory steps while maintaining high clinical accuracy.

Digital “one-visit” workflow: Utilizes full CAD/CAM integration by combining intraoral STL scans with CBCT 3D data. This enables the design of a fully customized surgical guide alongside the prefabricated Blondex appliance, allowing simultaneous TAD insertion and appliance delivery in a single clinical appointment.

### 3.1. Analog Workflow: Case 1

Case 1 concerns a 16-year-old female patient presenting a slightly concave profile, a significant finding in the sagittal plane (Figure 4). Frontal analysis showed a facial asymmetry characterized by a mandibular deviation to the left. In the vertical dimension, the patient was classified as normofacial, exhibiting balanced facial proportions. Lip competence was maintained at rest, while the smile analysis revealed a mild gingival exposure (gummy smile).

Intraoral inspection demonstrated good oral hygiene and overall dental health, wide buccal corridors, and complete permanent dentition.

The molar relationship was a full Class II bilateral. The canine relationship was characterized by a half-unit (half-cusp) Class II on both the right and left sides.

A slight maxillary constriction (contracted palate) was observed. Vertically and sagittally, the patient presented an increased overjet (6 mm) associated with an increased overbite (5 mm), suggesting a tendency toward an anterior deep bite or an increased vertical overlap.

The dental arches showed overall good alignment, except for mild mesiorotations of the mandibular central incisors (elements 3.1 and 4.1) and a 1 mm lower midline deviation to the left (Figure 4).

The orthopantomograph (OPT) showed no significant dental or osseous pathologies (Figure 5). The cephalometric analysis highlighted that the patient presented a skeletal Class I relationship, as indicated by an ANB angle of 3.8° and a Wits appraisal of 4.1 mm. The skeletal discrepancy was primarily related to a slight mandibular retrusion, while the maxilla was correctly positioned relative to the cranial base (SNA 82.2°, SNB 78.4°) and an accentuated curve of Spee (4 mm).

The growth pattern is hypodivergent, as evidenced by an FMA angle of 21.7°, suggesting a strong horizontal growth component.

A marked proclination of the maxillary incisors was recorded. The mandibular incisors showed a stable and correct inclination relative to the mandibular plane (IMPA 91.5°).

The treatment was performed with the Blondex appliance (Figure 6), combined with the freehand placement of two palatal miniscrews, without the aid of positioning guides. Two palatal miniscrews (1.7 mm in diameter and 8 mm in length) were used for skeletal anchorage. Initially, a double-track Blondex with TMA springs featuring horizontal loops was used to achieve initial derotation of the involved teeth. No anterior mandibular sliding was induced; instead, the treatment aimed at opening the occlusal plane with retrusion of point B. During the distalization and the opening of the occlusal plane, distalization of the upper arch was also performed. The patient presented a Brodie-type relationship in the premolar area, with an anterior mandibular lock that prevented forward mandibular movement. The initial apparent distalization, which simulated a complete Class II relationship, was partially resolved after mandibular unlocking and intrusion of the lower incisors, leading to an improvement in the interarch relationship.

Treatment duration, including distalization, alignment, leveling, and finishing, was 39 months (Figure 7). A Class I molar and canine relationship was achieved, with normal overjet and overbite values.

Superimposition of pre- and post-treatment cephalometric tracings revealed a 4° occlusal plane opening, retrusion of the pogonion, a stable ANB value (4°), and slight proclination of the lower incisors (IMPA = 98°) (Figure 8). Although the percentage of distalization was limited, a forward functional adaptation of the mandible was observed, contributing to the overall improvement of occlusion and the facial profile.

### 3.2. Mixed Workflow: Case 2

The patient was a 28-year-old female who presented a straight facial profile (Figure 9). Frontal analysis showed a slight facial asymmetry with a mild mandibular deviation to the left. In the vertical dimension, the patient was classified as normofacial, exhibiting balanced facial heights. Lip competence was observed at rest, and smile esthetics were within normal physiological limits.

Intraoral evaluation demonstrated good oral hygiene and overall dental health.

The molar relationship was characterized by a full Class II on the right side and a half-unit Class II on the left. The canine relationship presented a bilateral half-unit Class II.

The maxillary arch exhibited palatal tipping (endoinclination) of the posterior segments. Vertically, an increased overbite (6 mm), was detected. The overjet was 4.9 mm and, coincidence of the dental midlines was recorded.

Mild dental crowding was observed in both the maxillary and mandibular anterior segments.

The panoramic radiograph (OPT) revealed the agenesis or absence of the mandibular third molars (Figure 10).

The patient presented a severe skeletal Class II relationship, as indicated by a significantly increased ANB angle of 7.4° and a Wits appraisal of 8.3 mm. This discrepancy was primarily due to a retrognathic mandible (SNB 75°) relative to a normally positioned maxilla (SNA 82.4°).

The growth trend was classified as normodivergent with a tendency toward hyperdivergence, evidenced by an FMA angle of 26°.

Cephalometric findings showed a retroclination of the maxillary incisors, which is often compensatory in Class II division 2 patterns. Conversely, the mandibular incisors exhibited a degree of proclination (IMPA 95.2°; FMIA 58.8°).

Treatment was performed using the Blondex appliance in combination with the insertion of two palatal miniscrews (1.7 mm in diameter and 8 mm in length), planned bidimensionally on a lateral cephalometric radiograph (Figure 11). In addition, fixed orthodontic therapy was carried out (Figure 12). Initially, distalization mechanics were applied to the upper arch, while sequential banding was performed on the lower arch. Teeth 1.6 and 2.6 were used to apply Class III elastics in order to achieve lower uprighting on a 0.018 × 0.025-inch stainless steel archwire, with tip-back on the second molar to counteract the extrusive force produced by the Class III elastics.

This case was managed as an orthodontic compromise, since surgical correction would have been the ideal approach; however, the patient opted for a solely orthodontic, non-surgical treatment. Treatment duration, including distalization, alignment, leveling, and finishing, was 32 months (Figure 13). Superimposition of cephalometric tracings revealed an increase in the torque of the upper incisors and minimal loss of lower incisor inclination (IMPA = 98°) (Figure 14).

### 3.3. Digital “One-Visit” Workflow: Case 3

The patient was an 11-year-old male who presented a convex facial profile associated with facial asymmetry, characterized by a distinct mandibular deviation to the right (Figure 15). In the vertical dimension, the patient was classified as normofacial, exhibiting balanced facial heights. Competent lip posture was observed at rest, and smile esthetics were within physiological limits, showing adequate dental display and harmony.

Intraoral evaluation showed satisfactory oral hygiene with evidence of preventative sealants on the first permanent molars. The patient was in the late mixed-dentition phase.

The molar relationship was a full Class II bilateral, while the canine relationship presented an asymmetry, with a Class II on the right side and a Class I on the left.

The maxillary arch exhibited significant constriction, associated with a functional lateral shift (laterodeviation) to the right. Vertically, an increased overbite (4.7 mm) was recorded.

Severe anterior maxillary crowding was present, complicated by the significant distorotation of the central and lateral incisors (elements 1.1, 2.1, and 2.2), and an increased overjet (5 mm) was recorded. A mild crowding was recorded in the lower arch, considering the residual leeway space.

A panoramic radiograph (OPT) confirmed the late mixed-dentition stage, with no evident dental anomalies (Figure 16).

The patient presented a skeletal Class I relationship, indicated by an ANB angle of 2.1° and a Wits appraisal of 1.5 mm. However, both the maxilla and mandible appeared retrusive relative to the cranial base (SNA 75°; SNB 72.9°).

The growth trend was normodivergent, as evidenced by an FMA angle of 26.1°.

Cephalometric analysis confirmed a marked proclination of the maxillary incisors. Conversely, the mandibular incisors showed a stable position relative to the symphysis, with an IMPA of 89.6° and an FMIA of 64.3°. The patient’s parents were reluctant to consider an extraction-based treatment approach for their son. Nevertheless, extraction therapy remained a clinically viable alternative. Treatment was performed using the Blondex appliance combined with the insertion of two palatal miniscrews, planned tridimensionally on cone-beam CT, following a “one-visit” protocol—that is, both miniscrew insertion and appliance placement were completed in a single appointment (Figure 17). The palatal miniscrews both measured 2.0 mm in diameter. The right miniscrew was 9 mm long, whereas the left miniscrew was 8 mm long.

The CBCT examination was performed as part of the clinical diagnostic and treatment plan process, and was not performed solely for the purposes of this publication. Fixed orthodontic treatment was also carried out. Initially, TMA springs were used, and once the desired derotation of teeth 1.6 and 2.6 was achieved, they were replaced with stainless steel springs to allow distalization (Figure 18 and Figure 19).

Treatment duration, including distalization, alignment, leveling, and finishing, was 24 months (Figure 20). In this case, distalization mechanics were possible despite the presence of a complete Class II molar relationship, as the premolars and canines were in an end-to-end position due to forward tipping of the teeth secondary to delayed eruption of the upper canines. Superimposition of cephalometric tracings revealed a reduction in lower incisor inclination (IMPA = 85°) (Figure 21).

## 4. Discussion

Maxillary molar distalization has traditionally been performed with extraoral traction or intraoral appliances such as the Pendulum, Distal Jet, and Jones Jig. These approaches can produce clinically useful distal movement, but their effects may include anchorage loss, mesial movement of the anterior anchorage unit, incisor proclination, and, depending on the appliance and force system, distal tipping or molar rotation. Skeletal anchorage protocols address some of these limitations by transferring the anchorage demand from the dentition to palatal or buccal miniscrews. However, TAD-supported mechanics are not free of limitations: miniscrew stability, palatal bone anatomy, soft-tissue conditions, hygiene, insertion accuracy, and the magnitude and direction of the applied force remain clinically relevant factors. The Blondex appliance is conceptually comparable to other TAD-supported distalizers, but differs in its modular rail-and-activator configuration and in the possibility of combining distalization with derotation and selected vertical control within the same appliance architecture. Compared with the Beneslider, in which molar derotation may be difficult to control because of the rigidity and configuration of the steel sheet and shoe, the independent spring system of the Blondex may offer greater flexibility for managing side-specific rotational corrections. Similarly, although the Frog appliance also uses a 0.032-inch TMA spring, its single-spring configuration is intended to address both molars, whereas the Blondex employs separate springs for the right and left sides. The Blondex springs may also be modified or replaced according to the treatment objective, including a transition from rotational control to distalization mechanics. However, these comparisons are based on appliance design and clinical rationale; the present case series does not provide quantitative evidence demonstrating superior derotation, distalization, treatment efficiency, or mechanical reliability relative to the Beneslider, Distalslider, Frog, or other established systems. With respect to the clinical cases illustrated above, although the total treatment durations were 39, 32, and 24 months for Cases 1, 2, and 3, respectively, these intervals represent the complete orthodontic treatment and should not be interpreted as the duration or rate of active molar distalization. Standardized measurements of molar displacement in millimeters were not prospectively recorded for all cases; therefore, a reliable distalization rate could not be calculated. In Case 2, treatment was performed in an adult patient with a severe skeletal Class II discrepancy, as indicated by an ANB angle of 7.4°. Because the patient declined orthognathic surgery, the treatment represented a dentoalveolar camouflage approach involving orthodontic compensation rather than correction of the underlying skeletal relationship. The long-term stability of this correction cannot be determined from the present report, and may be influenced by the skeletal discrepancy, dentoalveolar compensation, retention protocol, and adjunctive mechanics used during treatment. Accordingly, this case should not be interpreted as evidence that the Blondex appliance can correct the underlying skeletal discrepancy or prevent relapse. Moreover, Case 3 involved an 11-year-old patient in the late mixed-dentition stage; therefore, the effects of ongoing growth and dental eruption must be considered when interpreting the treatment changes. In particular, the eruption and subsequent positional changes in the premolars and canines may have influenced the final molar relationship and the overall occlusal outcome. The potential effects of distalization on the developing dentition, as well as the stability of the achieved correction during subsequent growth, cannot be determined from the present report. Accordingly, the observed occlusal changes cannot be attributed exclusively to the Blondex appliance. In the clinical cases presented, this appliance represented an innovative system due to the introduction of several biomechanical and structural features, including:

- An anti-rotational mechanism aimed at improving the stability and control of dental movements.
- A modular design eliminating the need for conventional spring components, thereby simplifying clinical management of the appliance; for example, once the desired degree of distalization is achieved, the springs are removed, and a transpalatal bar can be applied to teeth 1.6 and 2.6. By connecting elastic chains between the Blondex activators and the bar hooks, intrusion movements can also be obtained (Figure 22).
- An intrusion system based on a guiding rail associated with support pins, designed to provide more precise biomechanical control.
- A fully integrated and highly versatile system, capable of offering both operational and economic advantages throughout the entire therapeutic pathway.

Despite the technical and clinical advantages illustrated by three representative cases, statistical generalization regarding the average rate of distalization or treatment duration remains constrained. Post-treatment evaluation in these cases relied primarily on 2D lateral cephalograms and intraoral scans to avoid unnecessary ionizing radiation exposure (ALARA principle). Consequently, fine 3D volumetric bone–root resorption analyses or cortical bone contact could not be quantitatively mapped via post-treatment CBCT.

The three cases illustrate the technical feasibility and workflow adaptability of the Blondex appliance. However, the present report does not provide quantitative evidence regarding chairside or laboratory efficiency, treatment costs, biological impact, or superiority over other distalization systems. These parameters require evaluation in prospective studies using predefined and standardized outcome measures.

## 5. Conclusions

This three-case series illustrates the use of the Blondex appliance as a palatal TAD-supported system for maxillary molar distalization in patients with different clinical presentations and treatment requirements. Its modular configuration may permit the combination of distalization with molar derotation and selected vertical control, while its design is compatible with analog, mixed, and fully digital workflows. The appliance may be considered in carefully selected patients requiring reduced reliance on dental anchorage, including cases in which unilateral or bilateral distalization and additional molar control are desirable. Nevertheless, the present report is descriptive, includes only three heterogeneous cases, and lacks a control group, standardized force measurements, systematic three-dimensional outcome assessment, and long-term follow-up. Therefore, the clinical observations should not be interpreted as evidence of superiority over existing distalization appliances. Larger comparative studies are needed to evaluate treatment effects, efficiency, adverse events, stability, and patient-centered outcomes.

## Figures and Tables

**Figure 1:**
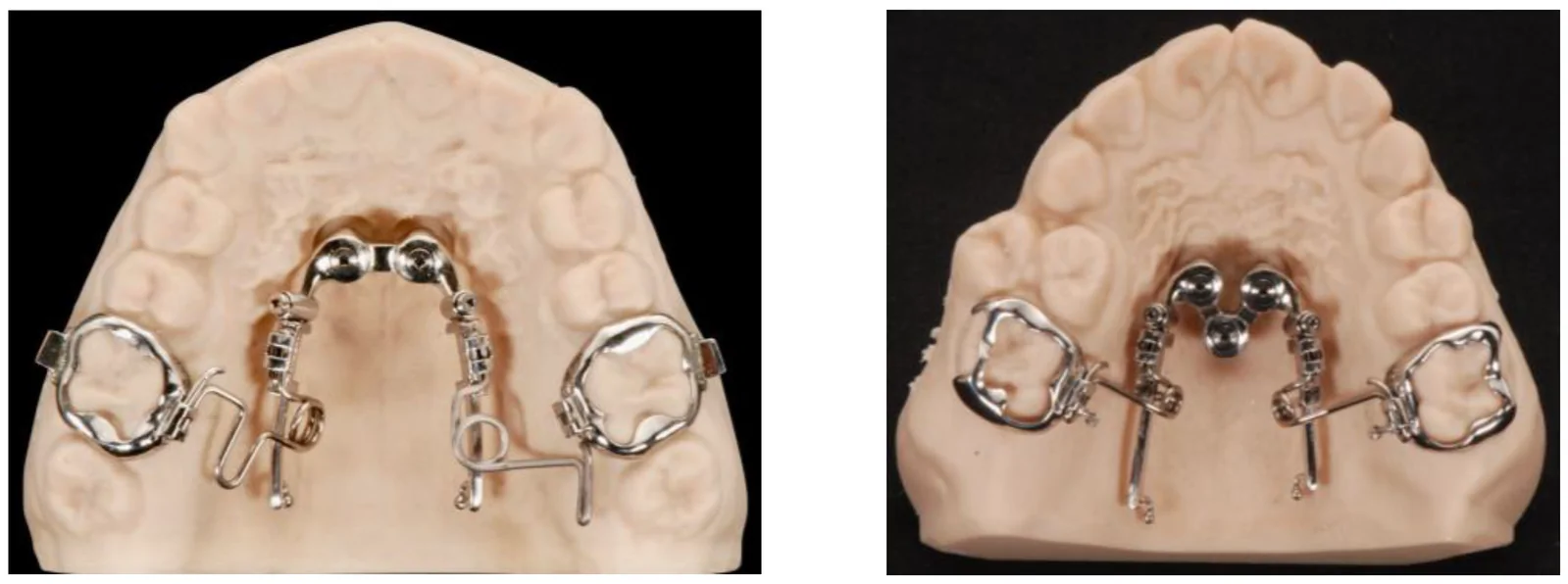
Blondex appliance with titanium–molybdenum alloy (TMA) springs on 26 and stainless steel (SS) springs on 16, on the left.

**Figure 2:**
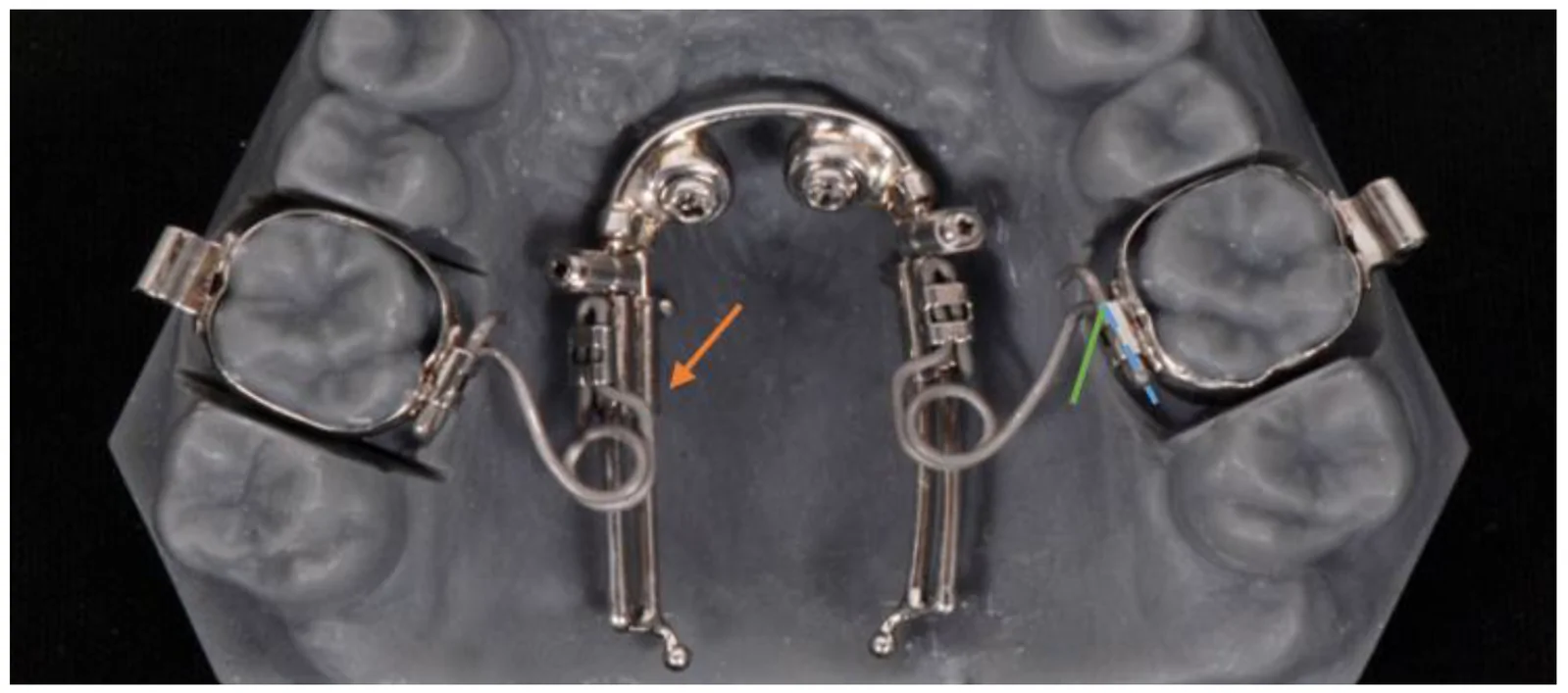
Two sliding arms in TMA with soldered cannulas. The orange arrow is the spot at which the spring can be opened to activate the appliance in order to facilitate expansion. On the other side, the dotted blue line indicates the passive position and green the active position when the spring is activated in an anti-rotational bend.

**Figure 3:**
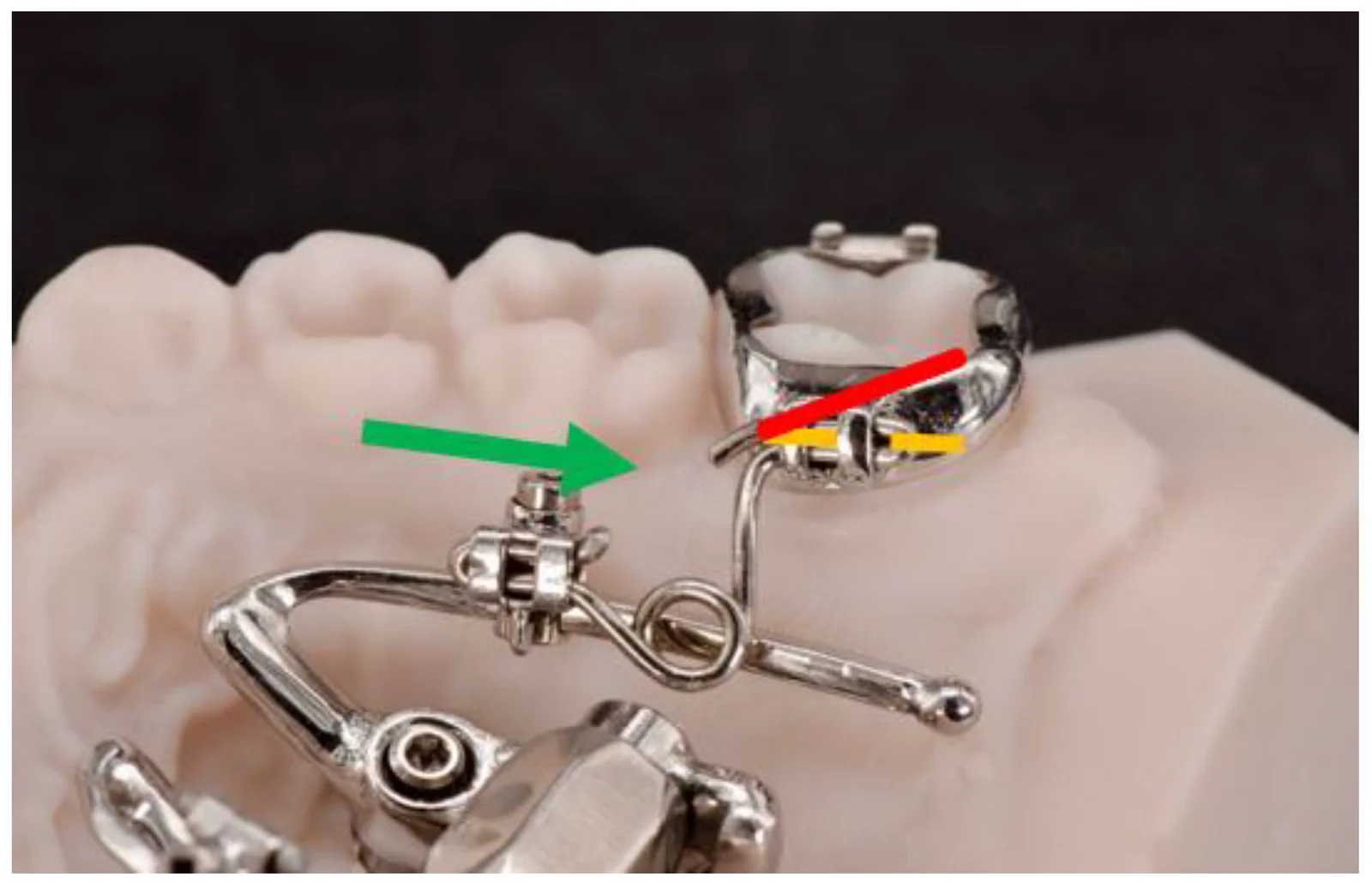
From the vertical plane, this figure shows the vertical activation of the shoe (yellow: passive; red: active). In green, the arrow indicates the direction of activation of the locking screw which allows for distalization. During clinical activation, the helix is open. This helix activation combined with the activation of the locking screw gives the distalization force.

**Figure 4:**
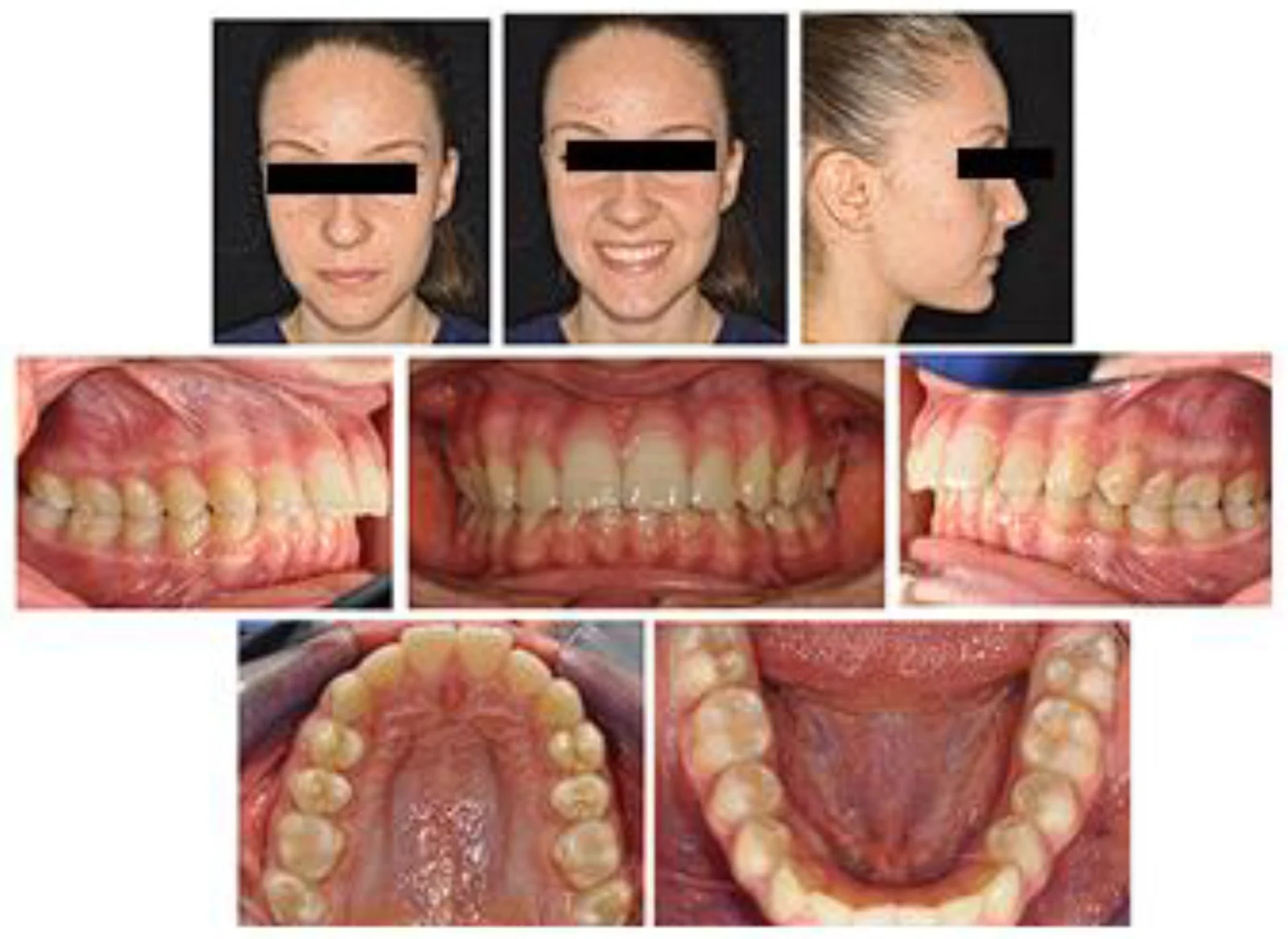
Extraoral and intraoral records before treatment.

**Figure 5:**
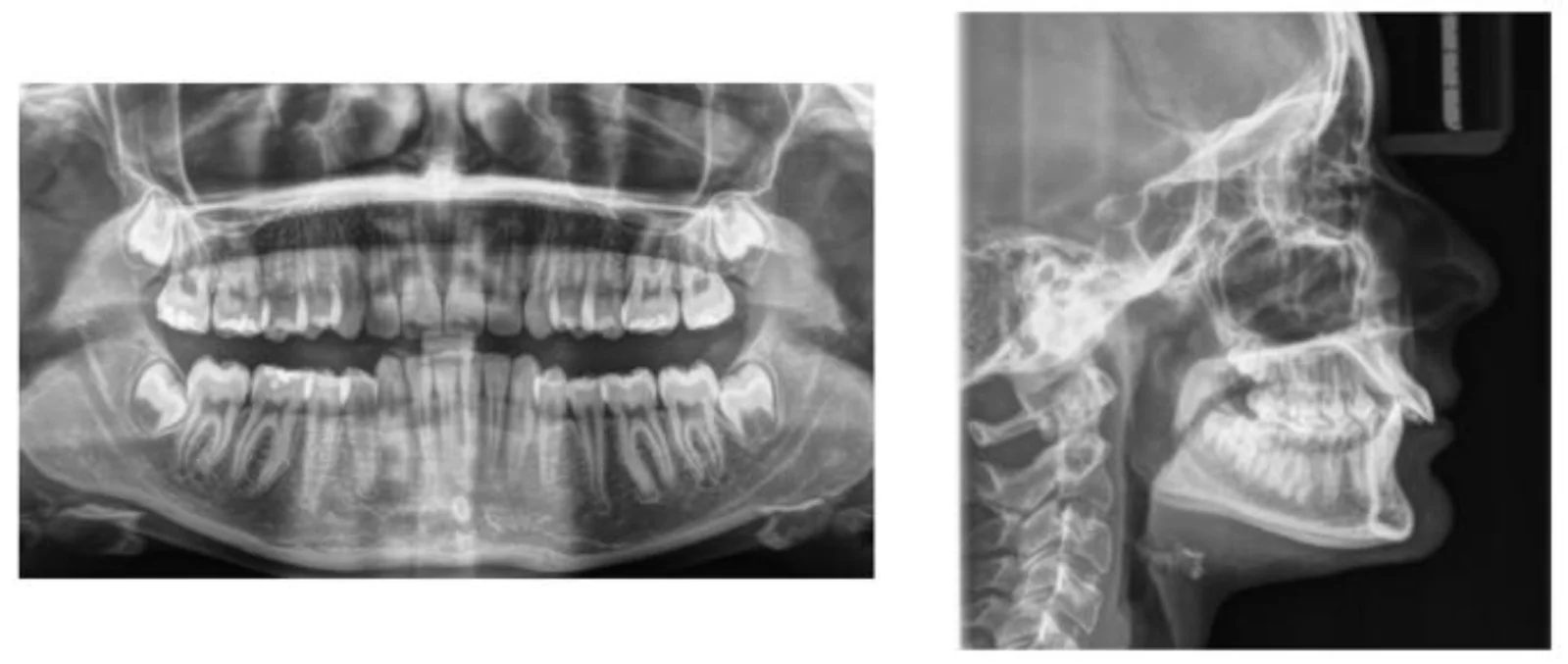
RX: OPT and lateral cephalometric X-ray before treatment.

**Figure 6:**
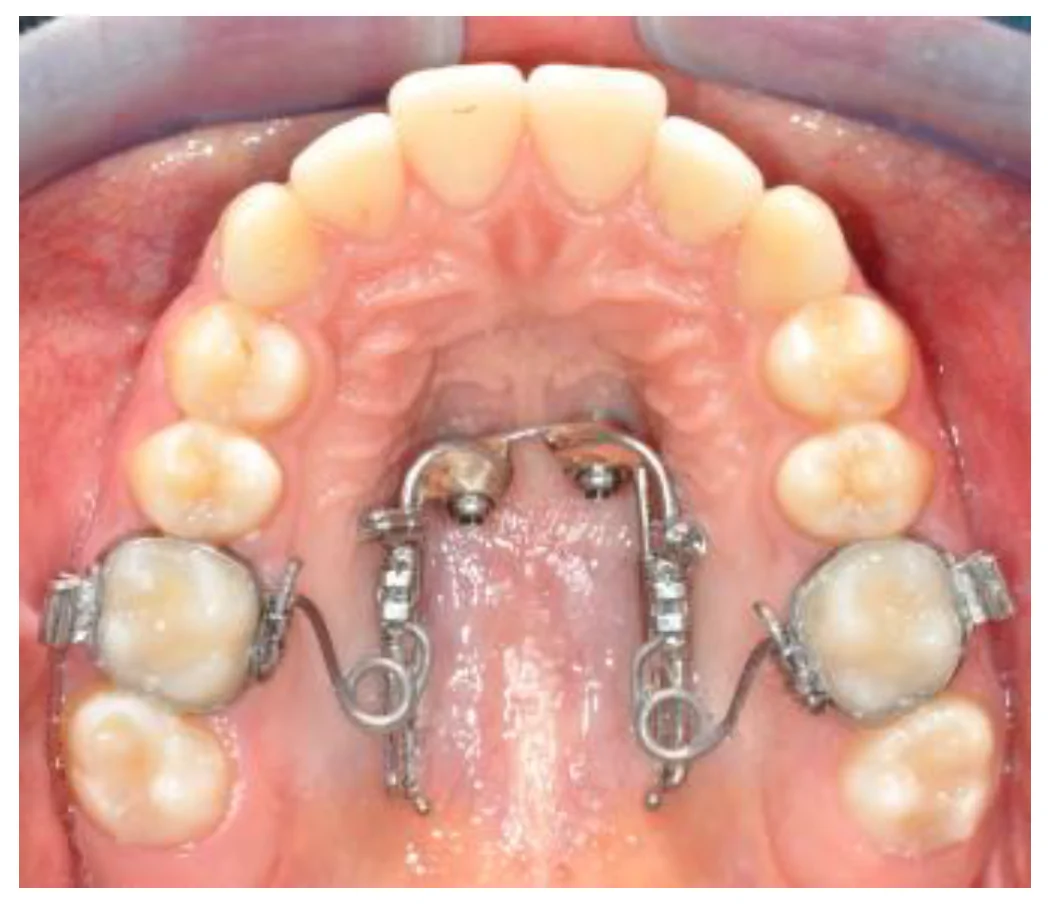
Blondex appliance—Case 1.

**Figure 7:**
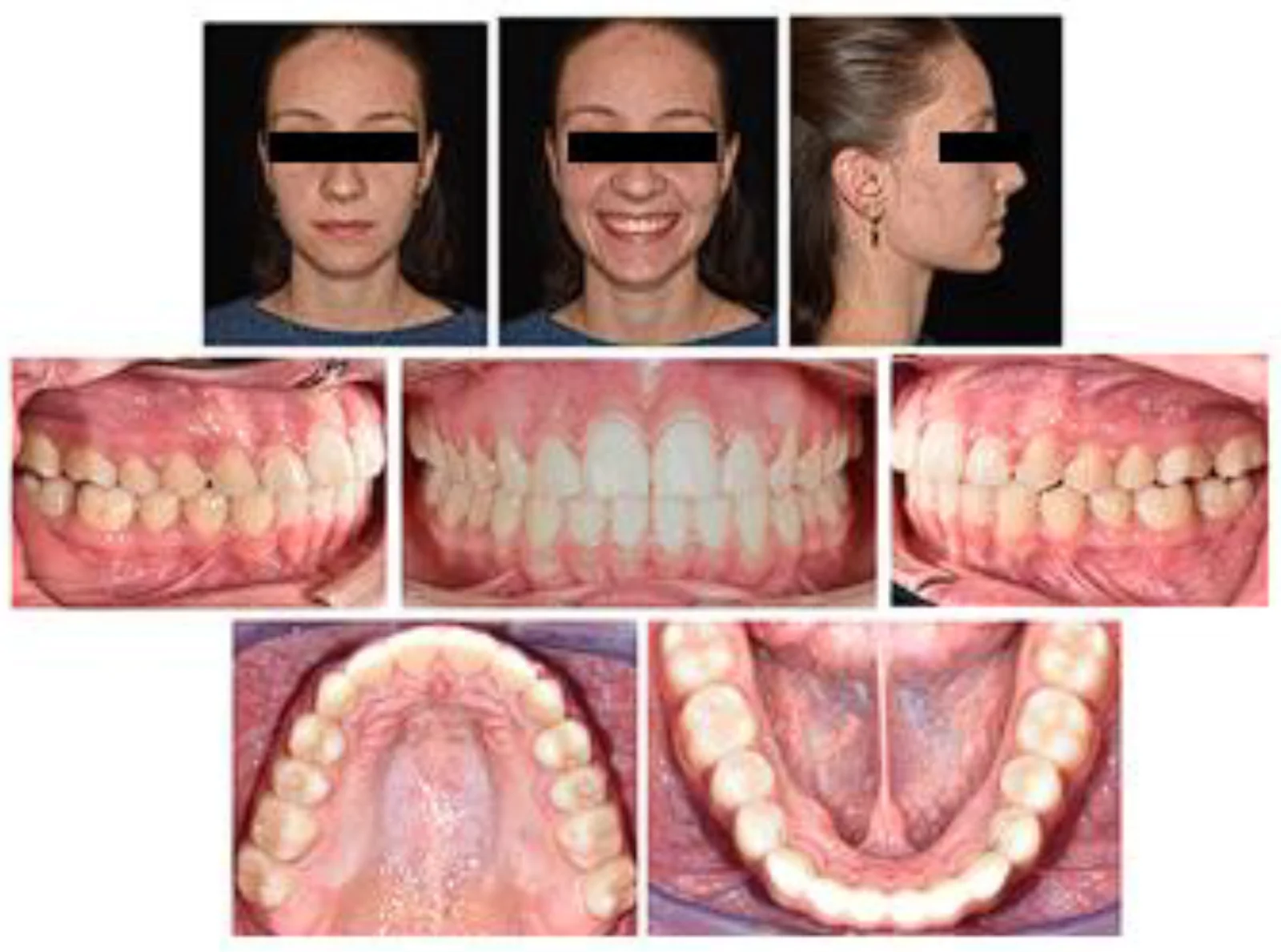
Extraoral and intraoral records after treatment.

**Figure 8:**
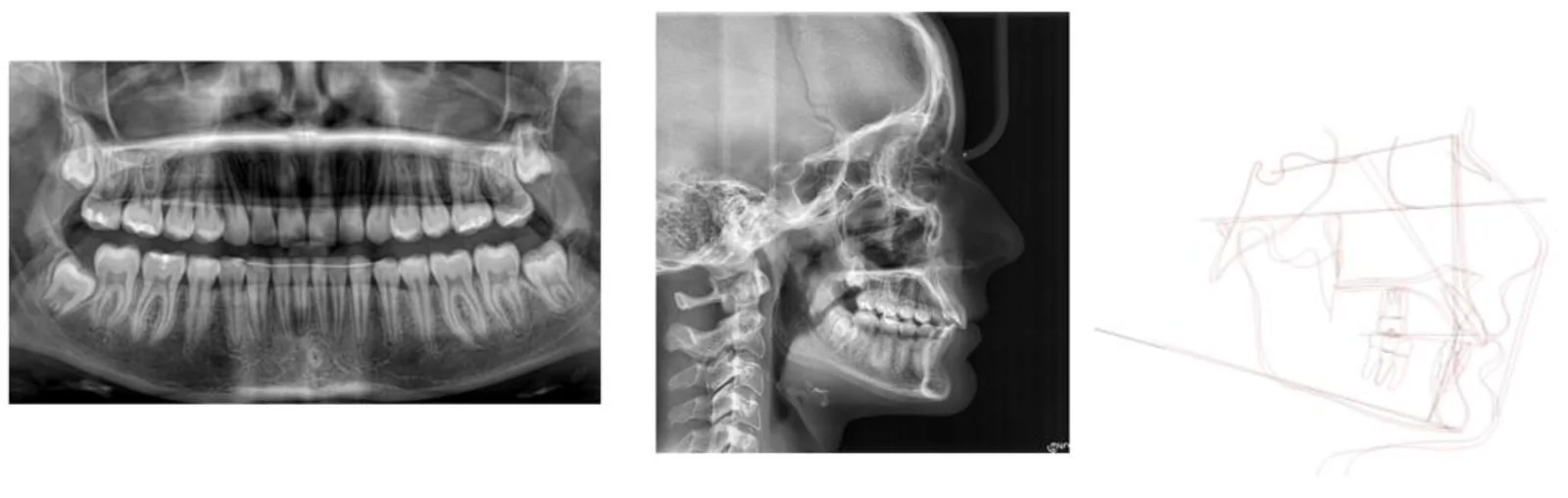
RX: OPT (**left**) and lateral cephalometric X-ray (**center**) after treatment and lateral cephalogram superimposition (**right**).

**Figure 9:**
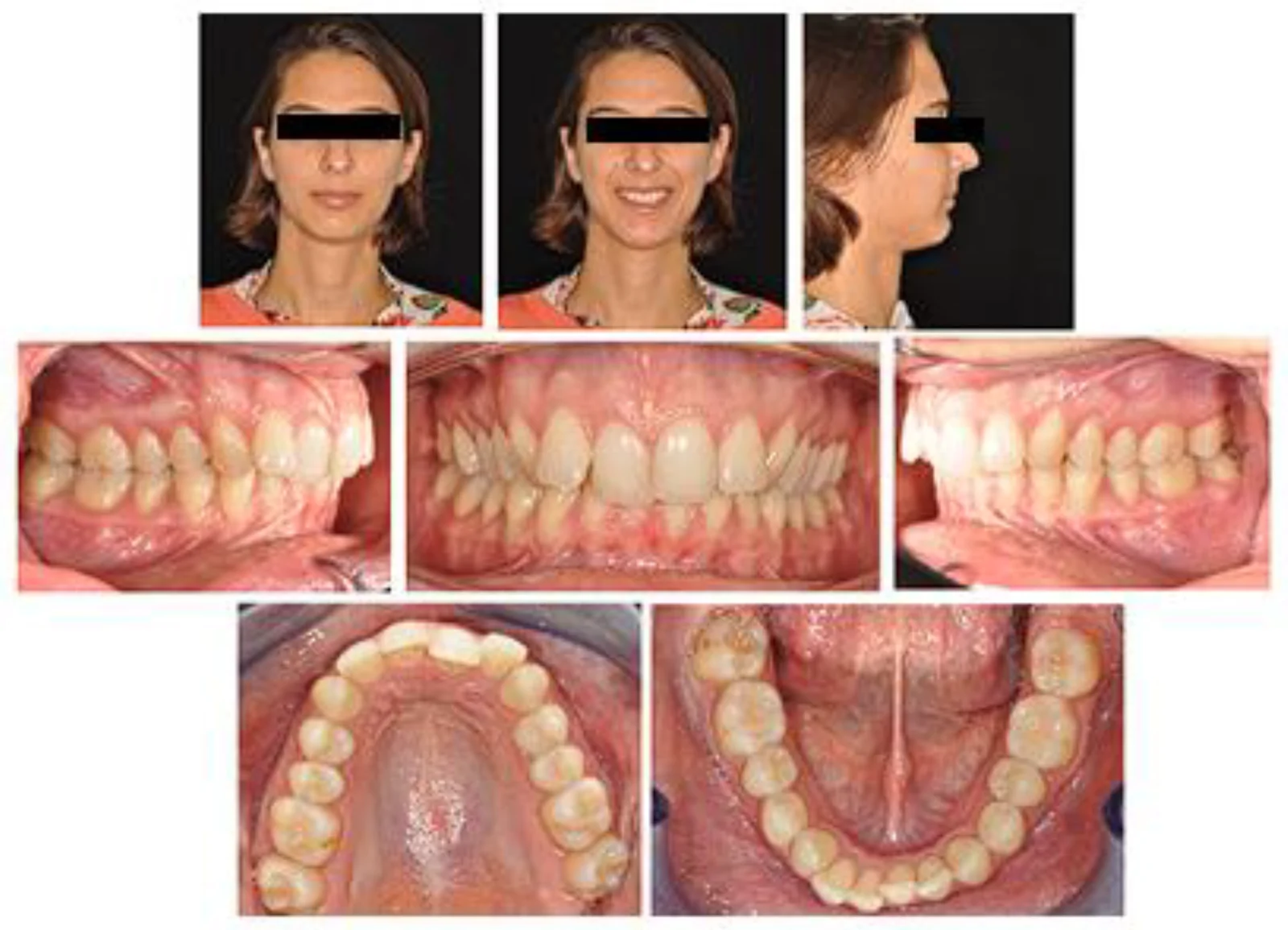
Extraoral and intraoral records before treatment.

**Figure 10:**
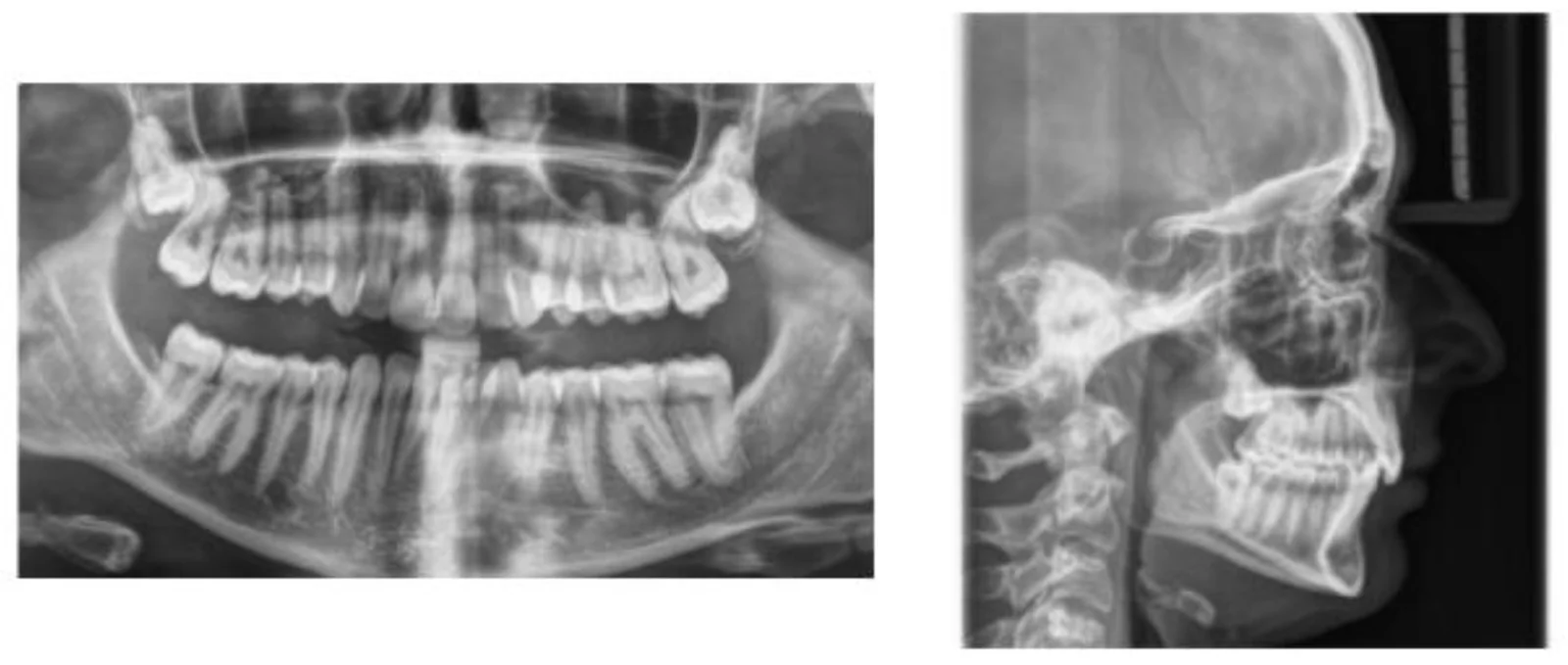
RX: OPT and lateral cephalometric X-ray before treatment.

**Figure 11:**
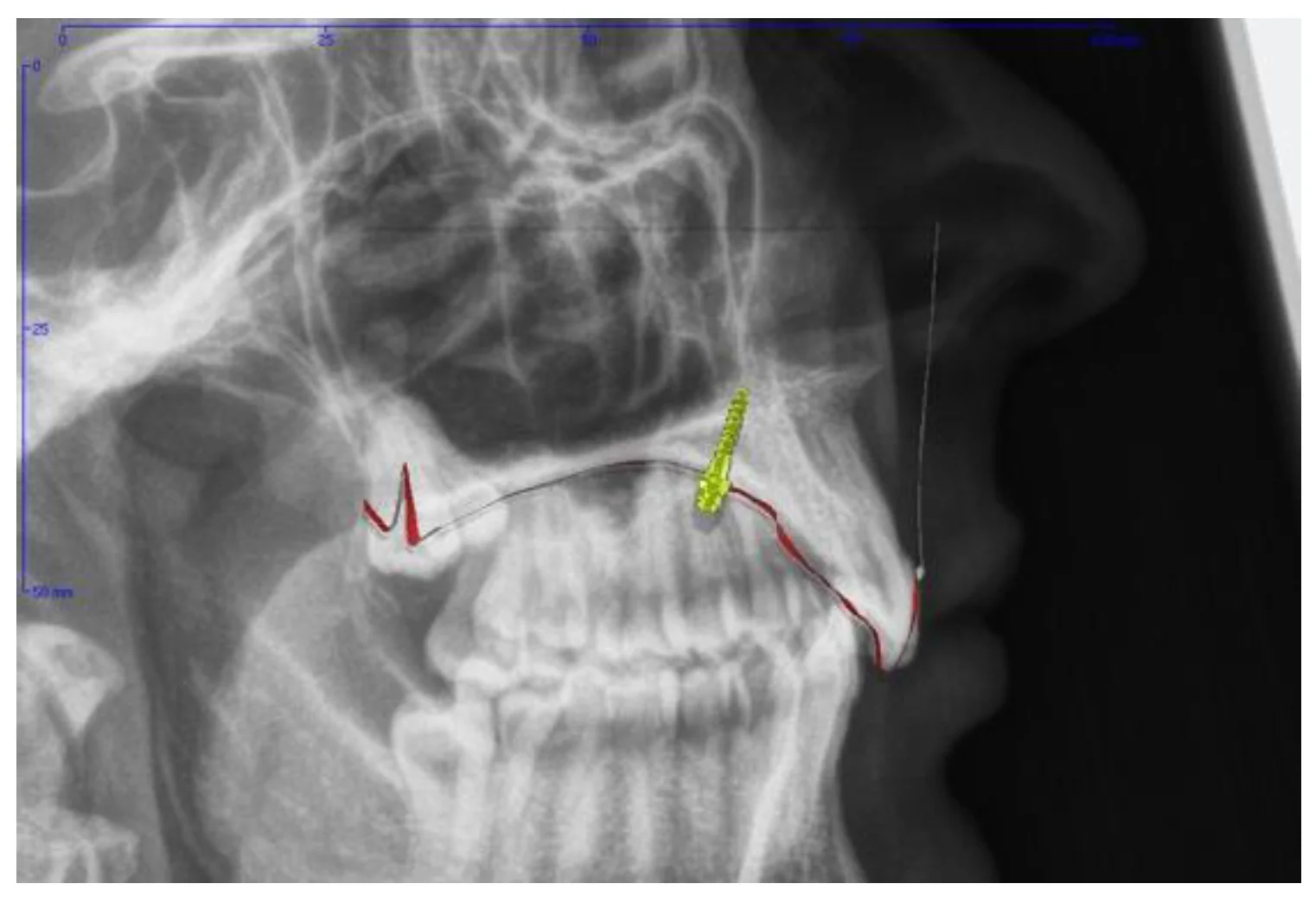
Bidimensional plan—Case 2.

**Figure 12:**
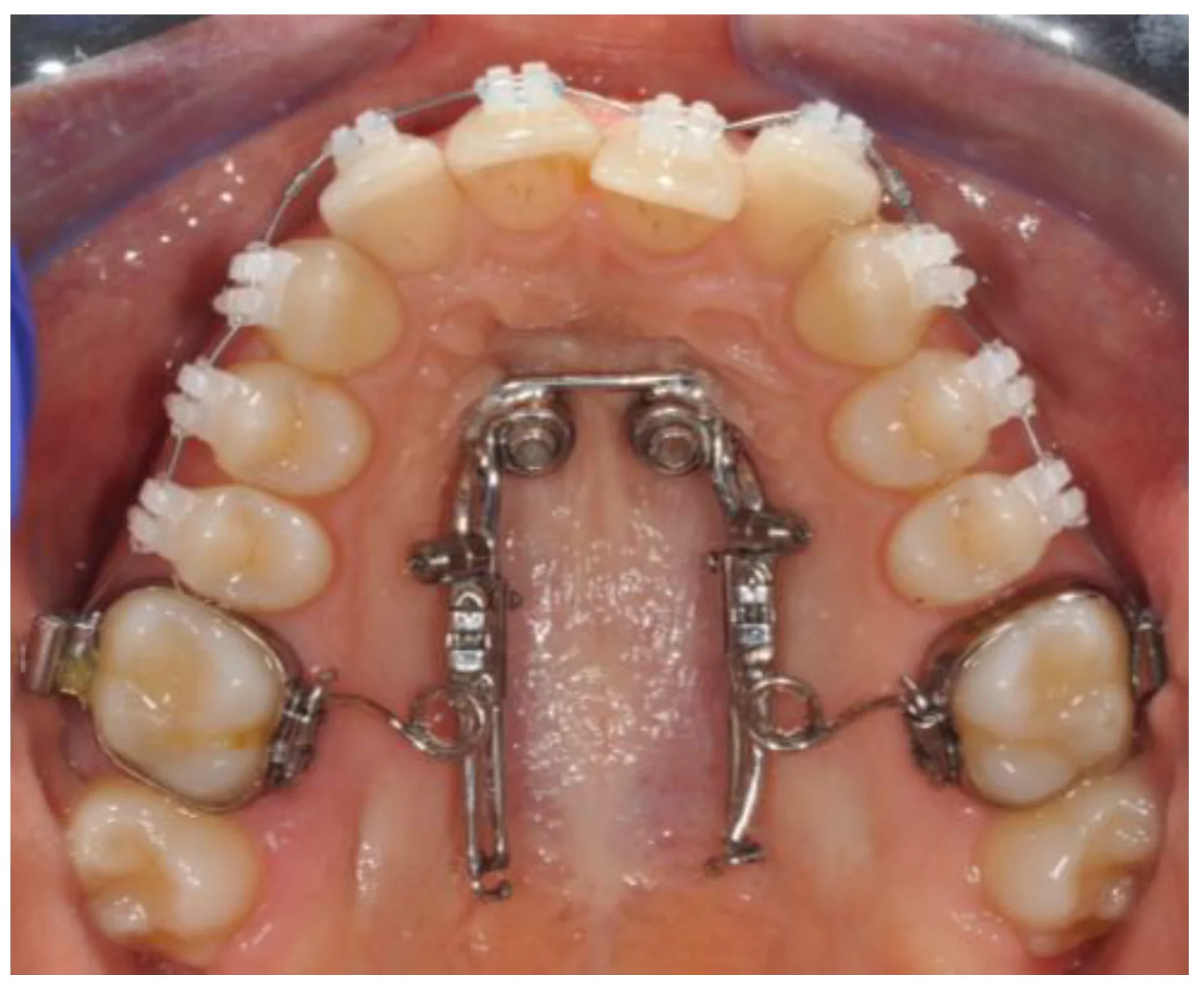
Blondex appliance—Case 2.

**Figure 13:**
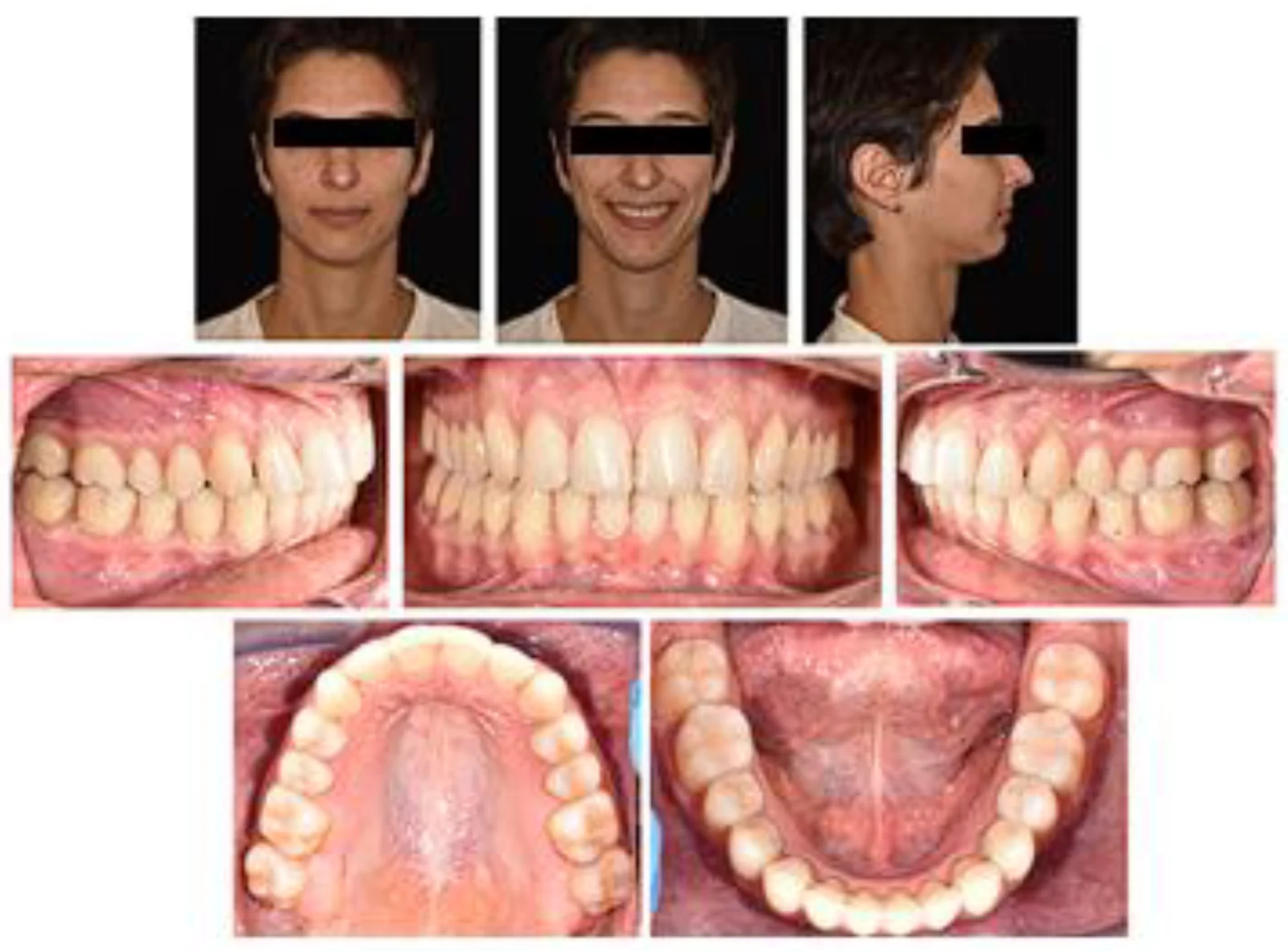
Extraoral and intraoral records after treatment.

**Figure 14:**
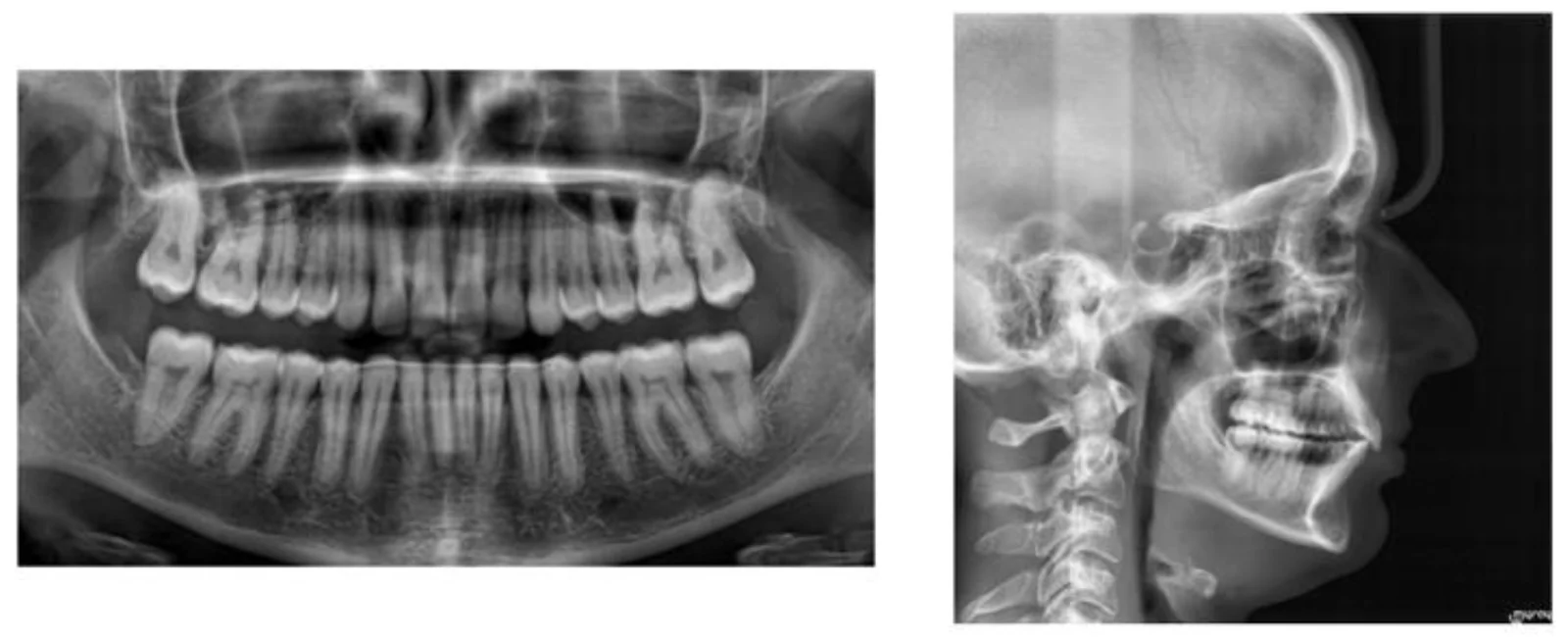
RX: OPT and lateral cephalometric X-ray after treatment.

**Figure 15:**
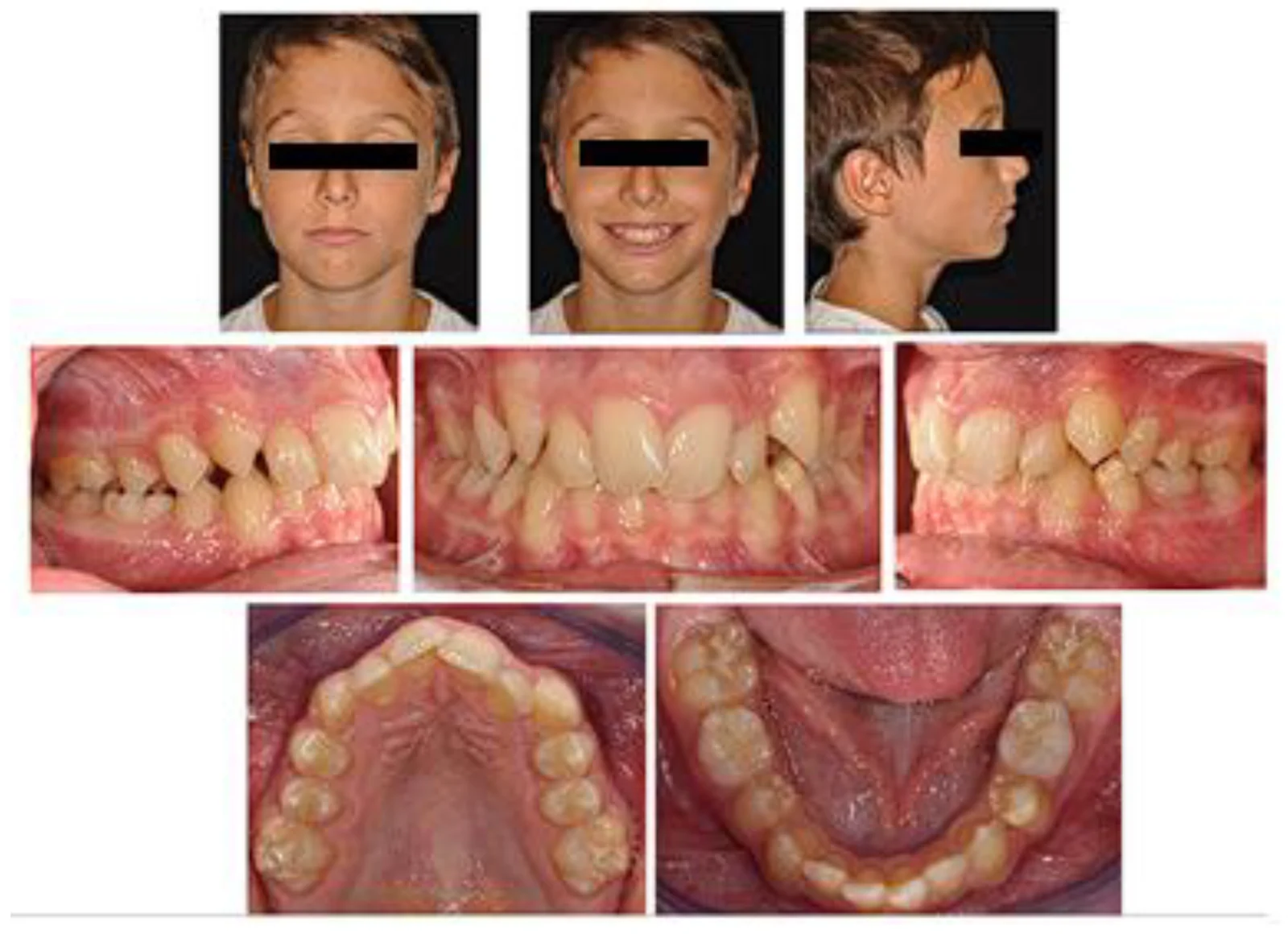
Extraoral and intraoral records before treatment.

**Figure 16:**
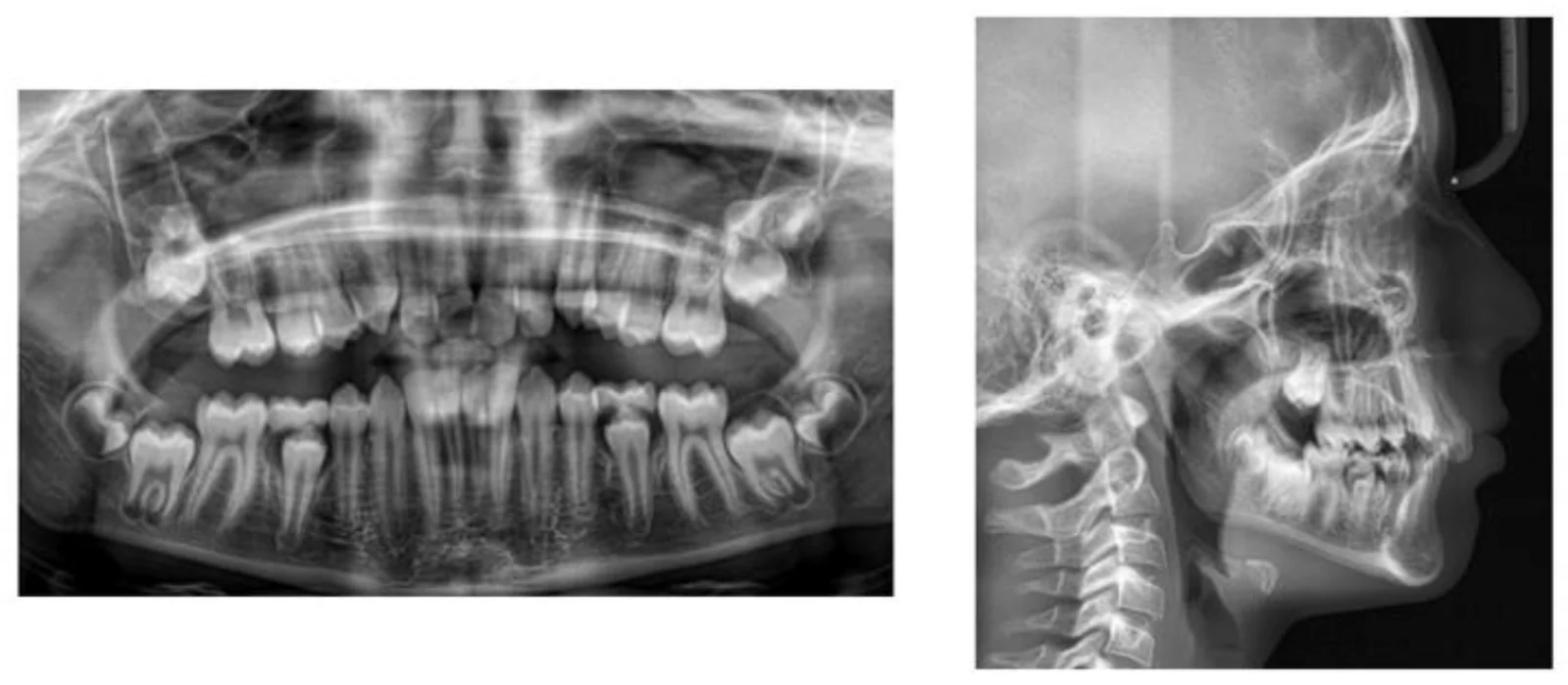
RX: OPT and lateral cephalometric X-ray before treatment.

**Figure 17:**
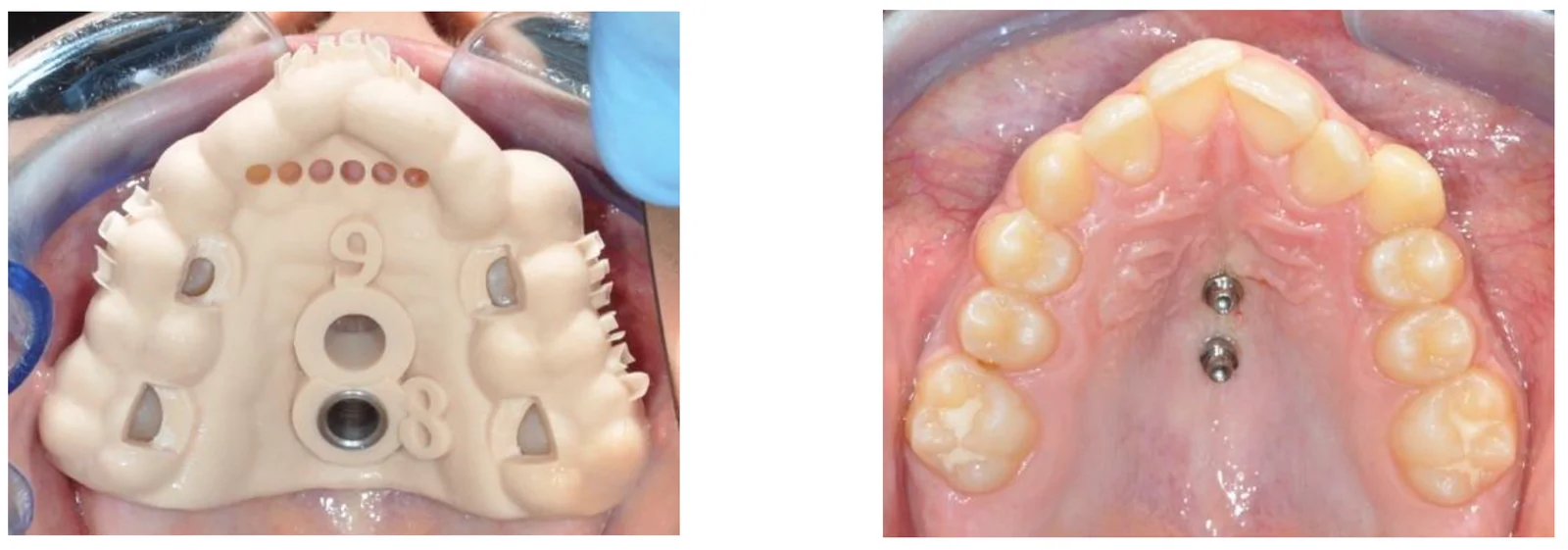
(**Left**) The surgical guide used to guide miniscrew insertion. (**Right**) The miniscrew inserted in the palate.

**Figure 18:**
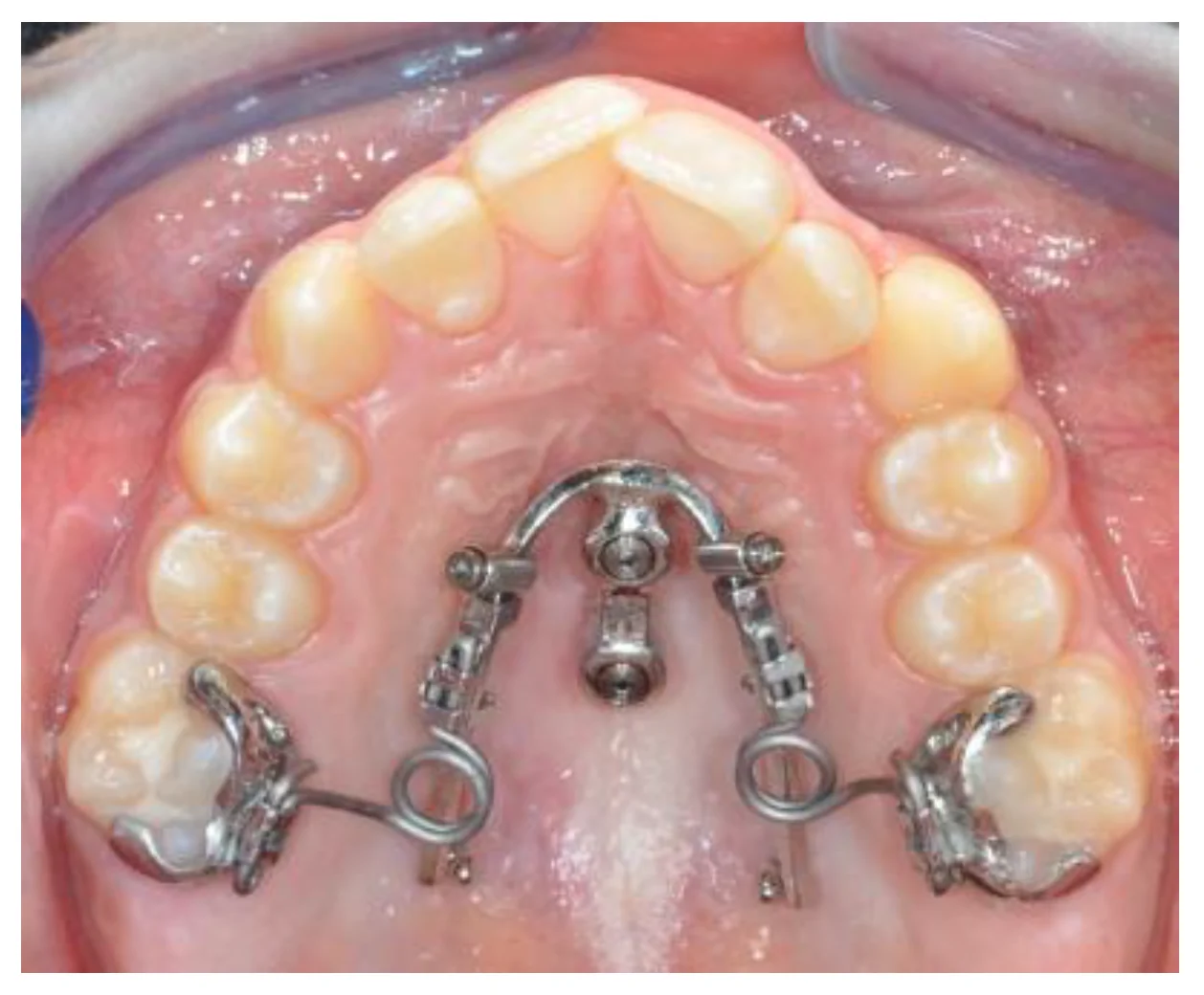
Blondex appliance with TMA springs—Case 3.

**Figure 19:**
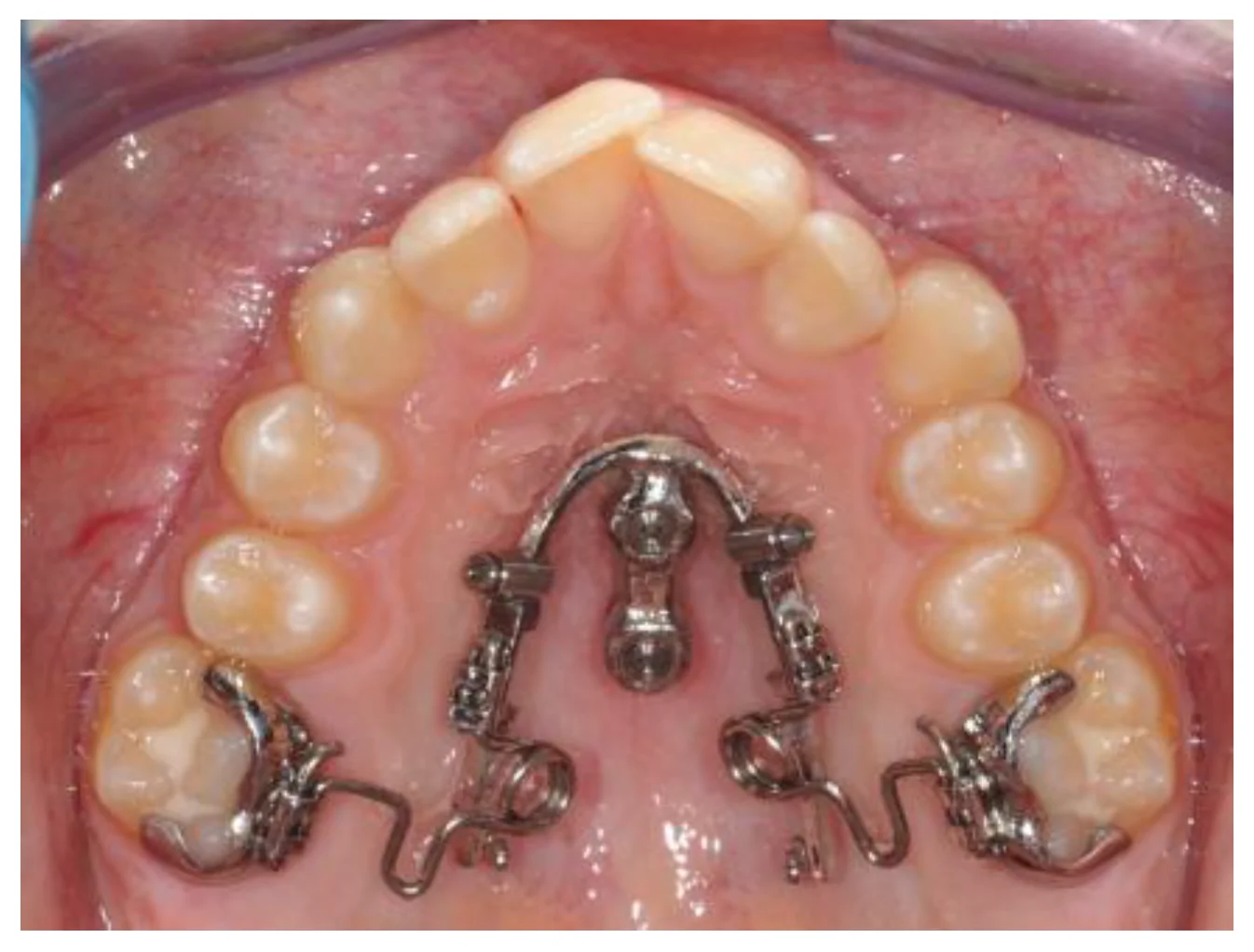
Blondex appliance with SS springs—Case 3.

**Figure 20:**
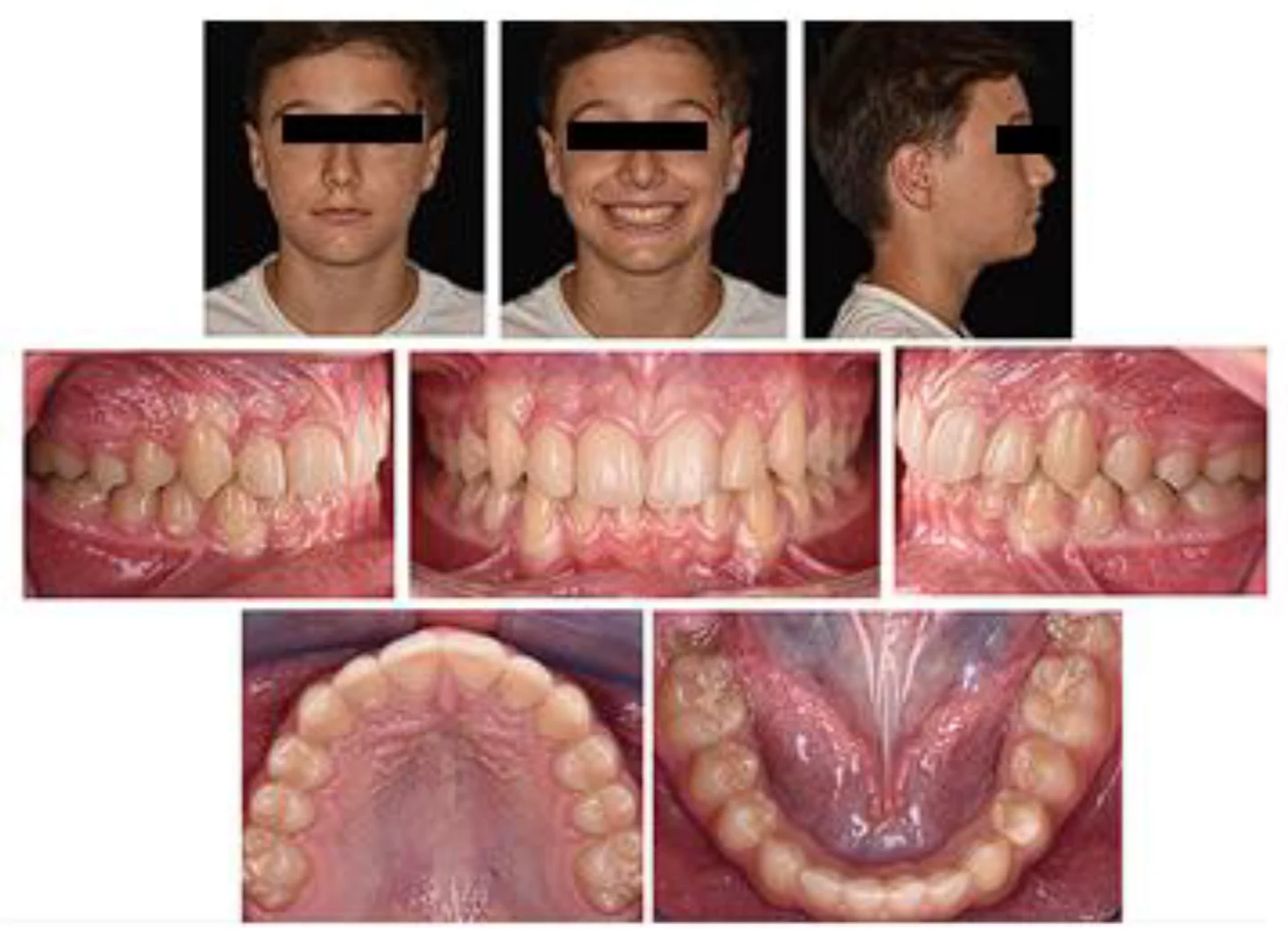
Extraoral and intraoral records after treatment.

**Figure 21:**
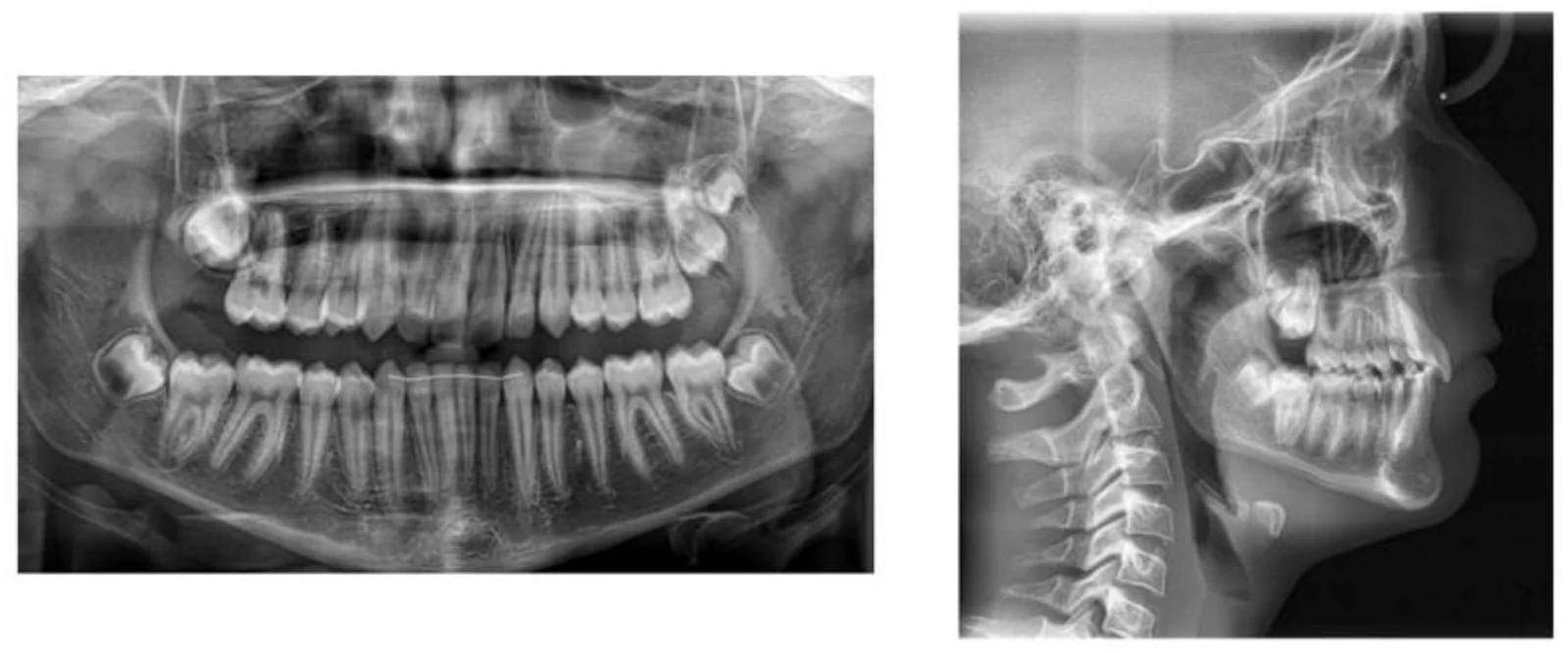
RX: OPT and lateral cephalometric X-ray after treatment.

**Figure 22:**
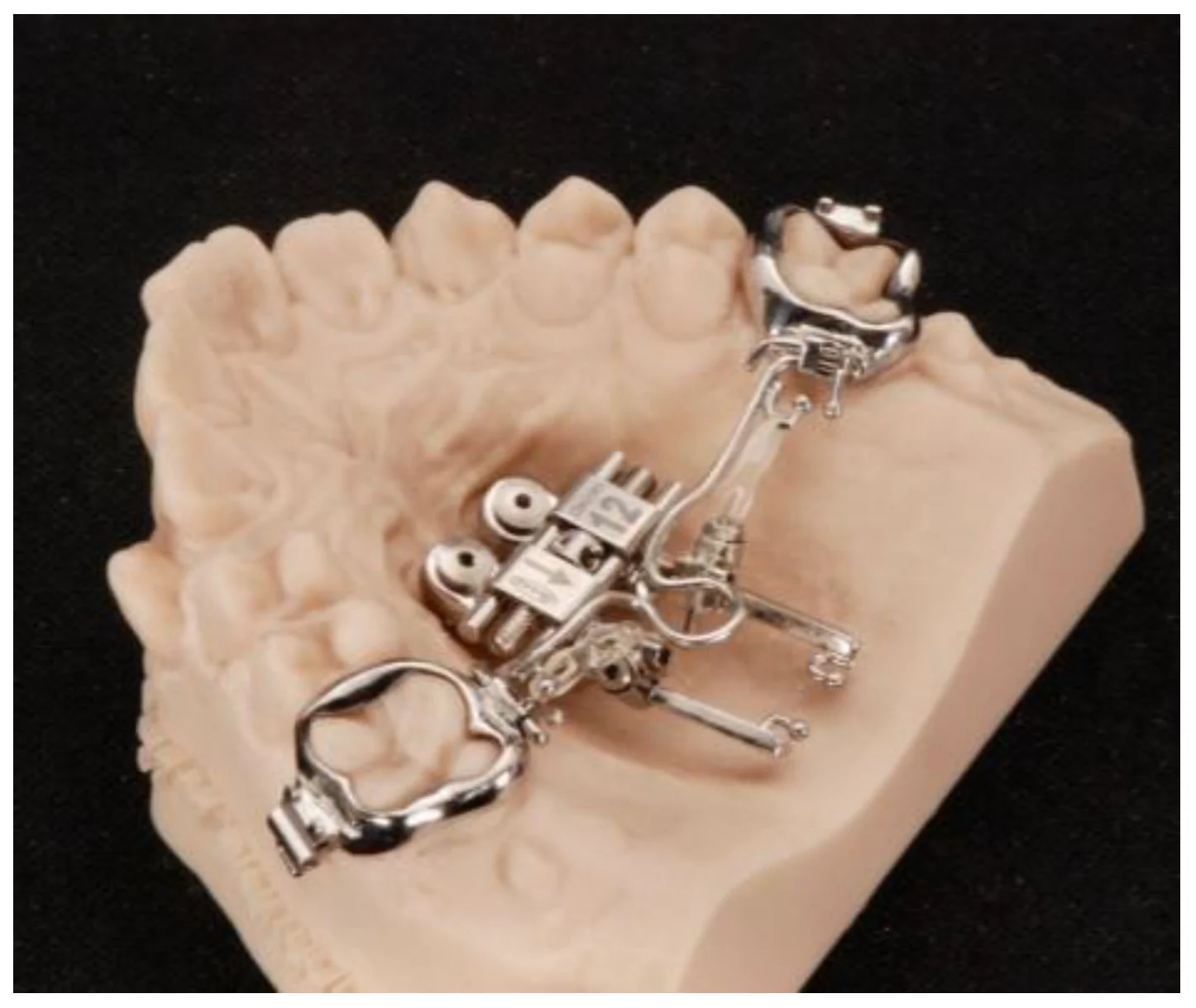
Blondex appliance modified with an expansion screw with a transpalatal bar and elastic chain for the molar’s intrusion.

**Table 1:** A descriptive comparison of the documented clinical and laboratory steps of the three workflows. It does not represent a comparative assessment of placement accuracy, treatment efficiency, cost, or biomechanical outcomes, as these parameters were not prospectively measured.

Workflow Parameter	Analog Protocol	Mixed Protocol	Digital “One-Visit” Protocol
Diagnostic and Planning	Conventional impression and plaster model	2D planning/bidimensional surgical template	3D-guided planning (CBCT + STL digital integration)
TAD Placement Approach	Freehand insertion	Semi-guided insertion	Fully guided insertion via CAD/CAM surgical guide
Surgical and Clinical Steps	Multiple clinical and laboratory sessions	Reduced laboratory phases	Single-visit procedure (simultaneous TAD placement and appliance insertion)
Appliance Fabrication	Manual adaptation on physical model	Hybrid fabrication (analog/digital)	Fully customized CAD/CAM design and manufacturing
Chairside Time and Efficiency	Standard chairside time	Not quantitatively assessed	Not quantitatively assessed

## Data Availability

The data presented in this study are available upon request from the corresponding author due to privacy restrictions.
